# CPT1C promotes the potential of gastric cancer ovarian metastasis through up-regulating fatty acid oxidation: CPT1C promotes the potential of gastric cancer ovarian metastasis

**DOI:** 10.3724/abbs.2022027

**Published:** 2022-03-03

**Authors:** Jianpeng Gao, Junquan Song, Yu Zhang, Zhenglun Zhu

**Affiliations:** 1 Department of Gastric Surgery Fudan University Shanghai Cancer Center Shanghai 200032 China; 2 Department of Oncology Shanghai Medical College Fudan University Shanghai 200032 China; 3 Department of General Surgery Shanghai Key Laboratory of Gastric Neoplasms Shanghai Institute of Digestive Surgery Ruijin Hospital Shanghai Jiao Tong University School of Medicine Shanghai 200025 China; 4 Department of Pathology Ruijin Hospital Shanghai Jiao Tong University School of Medicine Shanghai 200025 China

Ovarian metastasis (OM) of gastric cancer (GC), a common form of distant metastasis in female patients, frequently leads to the treatment failure and poor prognosis [1]. However, the molecular basis underlying the OM of GC remains largely unknown. With the recent advancement in transcriptome sequencing or RNA sequencing (RNA-seq) techniques, specific molecular features of metastatic malignancies have been uncovered [2]. In particular, RNA-seq of paired primary and metastatic lesions has identified metastasis-specific molecules which potentially serve as therapeutic targets and prognostic biomarkers [3]. In GC, the key promoters of peritoneal and hepatic metastasis have been identified by comparative transcriptome profiling of primary and metastatic lesions [ 4, 5]. Unfortunately, such techniques have not been applied in the distant OM of GC to date.

Consequently, in the present study we performed RNA-seq of primary tumors and matching ovarian metastases to identify key regulators underlying OM of GC. Our comparative analyses revealed the significant up-regulation of CPT1C, the protein-coding gene of a rate-limiting enzyme of fatty acid oxidation (FAO), in ovarian metastases over primary tumors. Subsequent clinical cohort studies indicated the selective up-regulation of CPT1C in the OM of GC. Functional assays showed that overexpression of CPT1C promoted the migration and invasion of GC cells and increased the expression of gastric cancer stem cell (GCSC) marker through up-regulating FAO rate. Our data suggest the unique significance of CPT1C in the OM of GC, which may serve as a novel therapeutic target in the future treatment.

Matched primary and ovarian metastatic lesions of 4 GC patients ( Supplementary Table S1) were submitted for high-throughput paired-end RNA-seq. As shown in Supplementary Figure S1A,B, a total number of 1088 differentially expressed genes (DEGs) were identified between primary and metastatic lesions, with 479 and 609 protein-coding genes up- and down-regulated in OM compared with primary GC, respectively. Functional pathway analysis revealed the cluster of glycosaminoglycan biosynthesis and fatty acid metabolism pathway in up-regulated genes in OM compared to primary GC ( Supplementary Figure S1C). Notably, it is identified that CPT1C, which encodes a key enzyme of lipid metabolism, is significantly up-regulated in ovarian metastases compared to primary tumors. CPT1C serves as the rate-limiting enzyme of FAO by essentially regulating transportation of fatty acid into mitochondria [6]. As the role of FAO in cancer progression has been increasingly recognized [7], we then focused on CPT1C to examine its significance in GC, especially in the distant metastasis to the ovary.

Initially, we examined the CPT1C expression on four paired tissues which were submitted for RNA-seq to validate our sequencing analysis. In line with the above analysis, CPT1C is up-regulated in ovarian metastases at both mRNA and protein levels when compared to those in primary tumors ( Figure 1A,B). Next, we investigated whether CPT1C is selectively up-regulated in distant metastasis of GC to the ovary rather than other common site such as peritoneum and liver. Additionally, we examined CPT1C expression in an independent clinical cohort which consisted of paired primary GCs and matching ovarian metastases ( *n*=21) and peritoneal and hepatic metastases ( *n*=15). Notably, the IHC staining score of CPT1C was significantly higher in the ovarian metastases than in the matching primary tumors ( *P*=0.010; Figure 1C), while the IHC staining scores of CPT1C were similar in primary tumors and matching peritoneal or hepatic metastasis ( *P*=0.289; Figure 1D). These results demonstrated the unique up-regulation of CPT1C in ovarian metastases compared to that in primary tumors, indicating the significance of CPT1C in GC metastasis to the ovary. Furthermore, we elucidated the role of CPT1C in GC progression through comprehensive analyses of cancer-OMICS databases, a similar strategy which was adopted in a previous study [8]. It was shown in UALCAN ( http://ualcan.path.uab.edu/index.html) that CPT1C expression was significantly higher in GC tissues than in non-tumor tissues ( *P*<0.01; Figure 1E). Notably, the differential expression of CPT1C was also observed between patients with and without lymph node metastasis ( Figure 1F) and between patients at pathological stage I and stage IV ( Figure 1G), implying its role in local and distant metastasis. Additionally, survival analyses in public databases showed that high expression of CPT1C was significantly associated with lower long-term survival rate ( Figure 1H,I). These results strongly suggested the correlation between CPT1C and GC severity in terms of cancer progression and metastasis.

**Figure 1:**
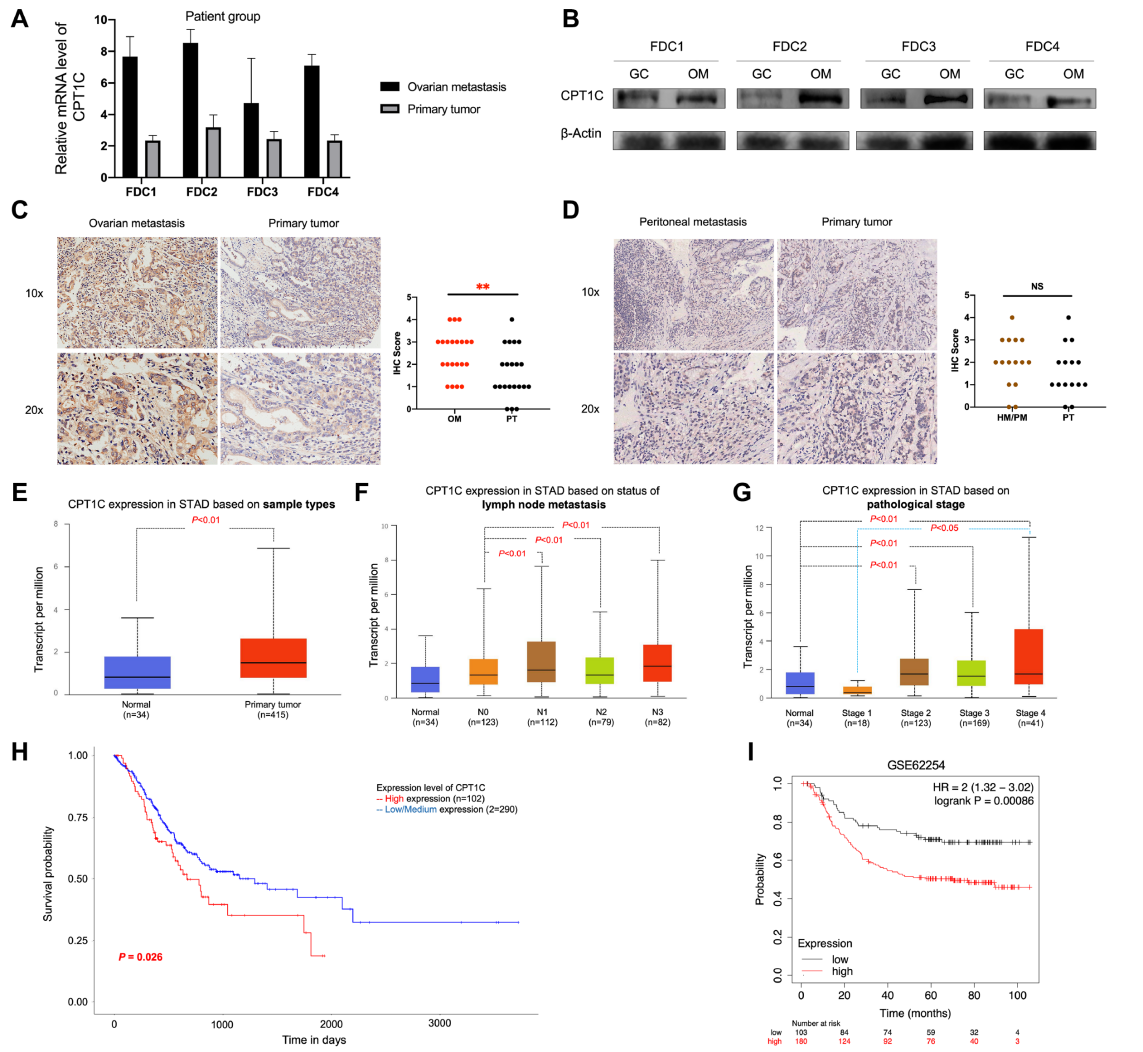
Figure1 Significance of CPT1C in GC and its ovarian metastasis (A,B) Validation of the differential CPT1C mRNA and protein expressions between 4 pairs of primary and ovarian metastatic tumors submitted for RNA-seq using qRT-PCR and western blot analysis, respectively. (C) IHC staining of CPT1C in the validation cohort showed the up-regulation of CPT1C in ovarian metastases compared to primary tumors (n=21; P=0.010). (D) IHC staining of CPT1C in the validation cohort showed similar expression of CPT1C between hepatic and peritoneal metastases and primary tumors (n=15; P=0.289). (E–G) Profiling of CPT1C expression with multiple criteria from TCGA-STAD database analyzed by UALCAN web-based tool. (E) Differential expression of CPT1C between primary GC and normal gastric mucosae. (F) Differential expression of CPT1C between patients with different status of lymph node metastasis. (G) Differential expression of CPT1C between patients at different pathological stages. (H,I) Kaplan–Meier plotters demonstrate the association between CPT1C expression and the clinical outcome of GC patients from TCGA-STAD and GSE62254 cohort of GEO database, respectively. TCGA, The Cancer Genome Atlas; GEO, Gene Expression Omnibus; and STAD, stomach adenocarcinoma.

To further assess the impact of CPT1C on the metastatic phenotype of GC cells, we conducted Transwell migration and invasion assays using AGS and HGC27 cell lines with enforced CPT1C expression ( Figure 2A,B). Since CPT1C is the rate-limiting enzyme of FAO, we also used the FAO assay to evaluate whether CPT1C could influence FAO rate. It was demonstrated that both FAO rate ( Figure 2C) and the number of penetrated GC cells ( Figure 2D,E) were significantly higher in the CPT1C overexpression group than in the empty vector-transduced groups. In addition, since gastric cancer stem cell (GCSC) is reportedly involved in both distant metastasis and metabolic reprograming of cancer cells [9], we also measured the mRNA expression levels of GCSC markers including CD44, EpCam and Lgr5 in both groups and found that all three markers were overexpressed in the ectopic CPT1C expression group compared to those in the empty vector-transduced group ( Figure 2F). Furthermore, we treated GC cells with Etomoxir, a FAO inhibitor, to determine whether CPT1C-mediated FAO is required for the malignant features of GC cells. Remarkably, CPT1C overexpression-induced increase of GC cell migration and invasion as well as GCSC marker expressions were both significantly inhibited after treatment with Etomoxir ( Figure 2G–I). Collectively, these results indicated the significance of CPT1C-mediated FAO in the pro-metastatic features of GC cells.

**Figure 2:**
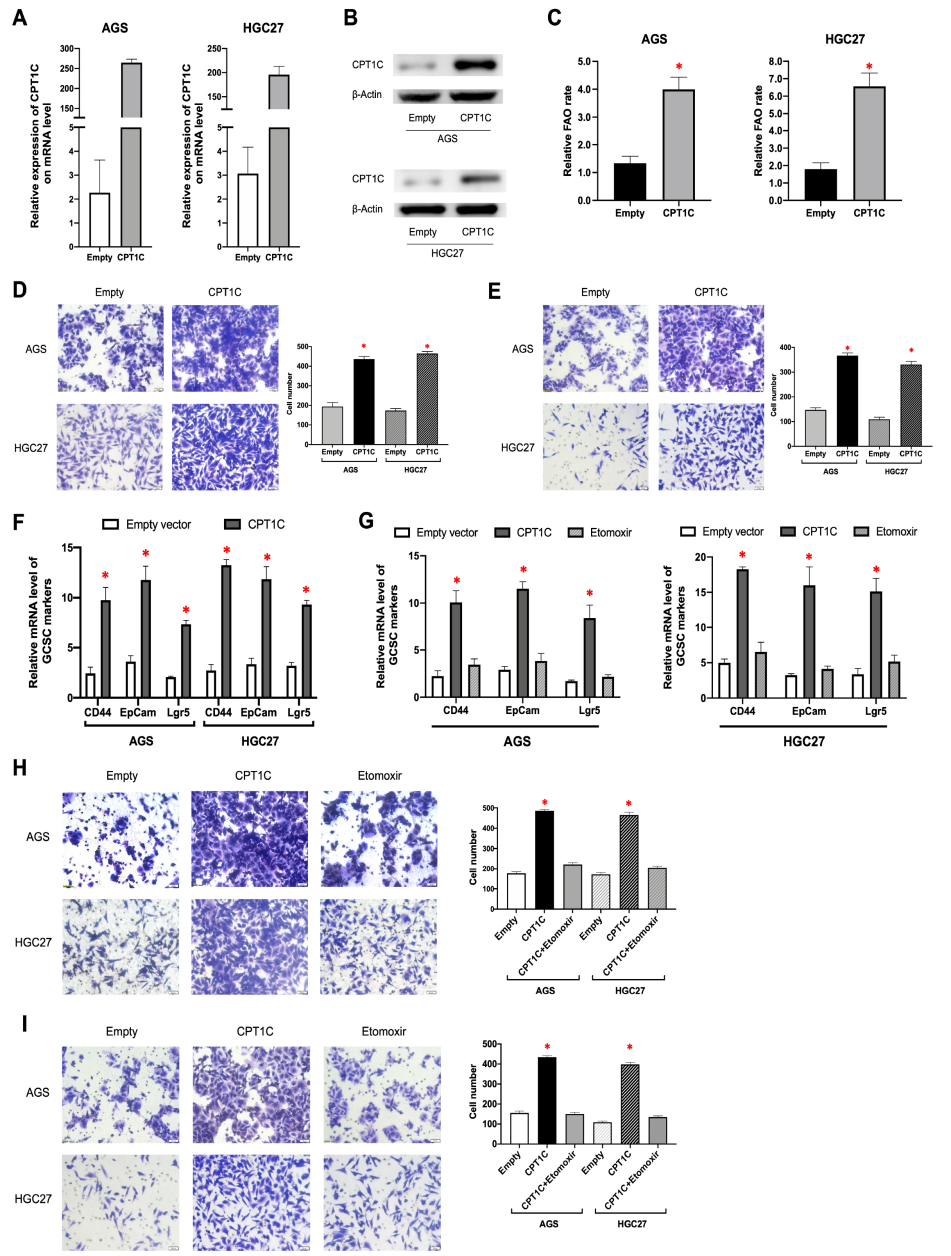
Figure2 Overexpression of CPT1C promoted GC cell migration and invasion and up-regulated GCSC markers expression via mediation of FAO (A,B) Ectopic CPT1C expressions in AGS and HGC27 GC cell lines validated by qRT-PCR and western blot analysis, respectively. (C) Overexpression of CPT1C significantly promoted the FAO rate of both AGS and HGC27 GC cell lines. (D) Overexpression of CPT1C significantly promoted the migration of both AGS and HGC27 GC cell lines. (E) Overexpression of CPT1C significantly promoted the invasion of both AGS and HGC27 GC cell lines. (F) Overexpression of CPT1C significantly up-regulated the mRNA expression levels GCSC markers (CD44, EpCam and Lgr5) in both AGS and HGC27 cell lines. (G) Etomoxir treatment significantly decreased the up-regulated expression of GCSC markers (CD44, EpCam and Lgr5) induced by forced expression of CPT1C. (H) Etomoxir treatment significantly inhibited the migration of GC cells with the forced expression of CPT1C. (I) Etomoxir treatment significantly inhibited the invasion of GC cells with the enforced expression of CPT1C. *P<0.05.

In summary, the comparative transcriptomic characterization of paired primary gastric and ovarian metastatic tumors identified CPT1C as a novel regulator of GC ovarian metastasis. Our clinical cohort study validated the selective up-regulation of CPT1C in ovarian metastases, while functional assays demonstrated that overexpression of CPT1C promoted GC cell migration and invasion and increased the expressions of GCSC markers through up-regulation of FAO rate. Our study reveals, for the first time, the significance of CPT1C in the distant metastasis of GC to the ovary and highlights the potential of CPT1C as a novel therapeutic target in the future treatment. Further investigations are required to clarify the molecular mechanisms underlying CPT1C-mediated FAO in promoting the distant metastasis of GC to the ovary by establishing an animal model to faithfully validate the impact of CPT1C on the ovarian metastasis of GC.

## Supplementary Data

Supplementary data is available at *Acta Biochimica et Biophysica Sinica* online.
